# Being Seen as a Unique Person is Essential in Palliative Care at Home and Nursing Homes: A Qualitative Study With Patients and Relatives

**DOI:** 10.1177/10499091241242810

**Published:** 2024-04-06

**Authors:** Katrin Kochems, Everlien de Graaf, Ginette M. Hesselmann, Saskia C. C. M. Teunissen

**Affiliations:** 1 Center of Expertise in Palliative Care, Julius Center for Health Sciences and Primary Care, 8124 University Medical Center Utrecht, Utrecht, The Netherlands; 2 Cancer Center, 8124 University Medical Center Utrecht, Utrecht, The Netherlands

**Keywords:** palliative care, personalized care, multidimensional needs, nursing home care, primary care, interview study

## Abstract

**Context:**

Incorporation of a palliative care approach is increasingly needed in primary care and nursing home care because most people with a life-limiting illness or frailty live there.

**Objectives:**

To explore patients’ and relatives’ experiences of palliative care at home and in nursing homes.

**Methods:**

Generic qualitative research in a purposive sample of patients with an estimated life expectancy of <1 year, receiving care at home or in a nursing home, and their relatives. Data is collected through semi-structured interviews and thematically analyzed by a multidisciplinary research team.

**Results:**

Seven patients and five relatives participated. Three essential elements of palliative care and their contributing factors emerged: 1) **be seen** (personal attention, alignment to who the patient is as a person, and feeling connected) 2) **information needs** (illness trajectory and multidimensional symptoms and concerns, and 3) **ensuring continuity** (single point of contact, availability of HCPs, and coordination of care). Patients and relatives experienced loss of control and safety if these essentials were not met, which depended largely on the practices of the individual health care professional.

**Conclusion:**

In both primary care and nursing home care, patients and relatives expressed the same essential elements of palliative care. They emphasized the importance of being recognized as a unique person beyond their patient status, receiving honest and clear information aligned with their preferences, and having care organized to ensure continuity. Adequate competence and skills are needed, together with a care organization that enables continuity to provide safe and person-centered care.

## Introduction

When a patient is faced with a life-limiting illness or frailty, the prioritization of a patients care shifts towards the quality of life and end-of-life considerations.^1^ Patients confronted with life-limiting illness, prefer to stay at home or a home-like surrounding.^2,3^

In the Netherlands (NL), 41% of individuals with palliative care needs die at home, and 27% in nursing homes.^4^ Home care is provided by general practitioners (GPs), district nurses (DNs) and nurse assistants.^5^ Other paramedical professionals can support and treat patients at home.^6,7^ As multiple care-organizations are involved, multiprofessional consultation is not regulated. PATZ groups (palliative care at home) are available, in which professionals can collaborate on a voluntary basis.^8^ When patients are unable to stay at home due to intensive care needs, nursing home care is available where care is primarily provided by nurse assistants, supported by nurses, in collaboration with physicians and other professionals.^9^ Multiprofessional consultations are held for every patient at least twice a year.^10^

Palliative care aims to improve the quality of life and dying through management of physical and psychological symptoms and social and existential concerns.^1^ Assessment and management should be considered as a multidimensional experience in all four dimensions and is provided through an interprofessional collaboration of health care professionals (HCPs).^11^ Patients’ and relatives’ values, wishes and needs should be the basis on which decisions are made.^11^

Recognition and treatment of symptoms,^12,13^ the associated communication,^12,14^ as well as guidance in shared decision-making and psychological, social, and spiritual support, are essential for patients and relatives to experience good palliative care.^12^ In practice, palliative care is often inadequate for patients cared for at home and in nursing homes as care is not integrated or integrated too late, symptoms and concerns are not recognized or uncharted, and many symptoms remain under or over-treated.^15-18^ Patients and relatives often experience deficiencies in palliative care competence, collaboration, percon-centered focus and access to HCPs.^19-22^ There is room for improvement, specifically in addressing the needs of patients andrelatives.^15,23^ A clear understanding of the experiences is essential for optimal palliative care in practice and integral to the quality of care,^24,25^ taking into account several patient characteristics, such as age, gender, educational level, place of residence and patients’ diagnosis, who have an effect on how care is experienced.^26-29^ The aim of this study is therefore to explore the experiences of patients and relatives with palliative care at home and in nursing homes.

## Methods

### Design

A generic qualitative study was conducted using inductive thematic analysis to explore the experiences of patients and their relatives with daily palliative care practices.^30^ The study followed the Consolidated Criteria for Reporting Qualitative Research (COREQ) for reporting.^31^

### Participant Selection

A purposive sample of patients with an estimated life expectancy of less than one year who received care at home or in a nursing home were eligible for this study. If the patient could not be interviewed or was already deceased, the primary contact person (referred to as 'relatives') was interviewed. Maximum variation was sought with respect to sex, age, education, place of residence, and patients' diagnoses. The exclusion criteria were cognitive impairment and language barrier for both patients and relatives. Participants were identified and recruited through nursing homes, DNs and GPs in the central region of the NL.

### Data Collection

Data were collected between May and October 2019 through semi structured interviews by one researcher (KK) at participants’ preferred locations. A topic list (supplementary material 1) was developed based on the Netherlands Quality Framework for Palliative Care,^11^ and scientific literature. A multidisciplinary team of experts and one pilot interview, not included in the analysis, validated the topic list. The topics covered were: experienced care, identification of multidimensional symptoms and concerns, decision-making, communication with HCPs, continuity of care, the burden on relatives, and care recommendations. All interviews began with a broad opening question: 'How are you doing now, and what was the last period (last month) like for you?' to build rapport. Non-verbal expressions were noted in memos. After each interview, a methodological report was written consisting of 1) a brief description of the participant, 2) description of interview location and environment, 3) the course of the interview, 4) reflections on the questionnaire, and 5) other information. Member checks occurred during the interviews by summarizing the participants' responses. Data collection and analysis alternated, allowing for adjustments to the topic list as new insights emerged. Data collection continued until saturation was reached on the main themes.^32^ All interviews were audio-recorded, transcribed verbatim, and anonymized. In addition, respondents’ demographic information was collected.

### Data Analysis

A thematic analysis was performed following the method described by Braun and Clarke.^30^ The data were analyzed within a research team to ensure research triangulation, comprising of an epidemiologist with qualitative research training (KK), a senior qualitative researcher (EdG), a nurse practitioner in palliative care and palliative care consultant (GH), and a senior researcher who is an oncology nurse (ST). After the initial three interviews, meaningful segments were identified through open coding independently by two researchers (KK and EdG). The findings were discussed, and preliminary codes were established. Subsequently, new participants were purposively included. This process repeated after the subsequent three interviews. After initial coding of the first six interviews, a consensus-based coding tree of main and subcategories, was developed by the research team. Subsequently, axial coding was conducted using NVivo 12.^33^ In the analysis of the ninth interview, no new themes or additional meanings emerged. To ensure data saturation, three additional interviews were conducted. Demographic data were summarized using Microsoft Office Excel, version 2016.^34^

### Ethical Considerations

This study was conducted following the principles of the Declaration of Helsinki^35^ and the General Data Protection Regulation.^36^ The Utrecht Medical Research Ethics Committee classified this study as exempt from the Medical Research Involving Human Subjects Act (WAG/mb/19/010 438). All participants received written and verbal information and informed consent was obtained before each interview.

## Results

### Participants Characteristics

Twelve participants were included, seven patients and five relatives (Table 1) of whom 58% lived at home and 42% in a nursing home. Most participants were female (75%), aged 69 (±12) on average and had an intermediate vocational education (67%). The patients’ primary diagnoses were cancer (58%), cerebral infarction (17%), Amyotrophic Lateral Sclerosis (8%), Parkinson (8%), and heart failure (8%). Interviews were performed at home or a nursing home and lasted 87 minutes on average (43-125).

**Table 1.** Participants Characteristics (n = 12).

	Patient (n = 7)		Relative (n = 5)		Total (n = 12)	
Gender, n (%)						
Female	4	(57)	5	(100)	9	(75)
Male	3	(43)	0	(0)	3	(25)
Age, mean years (±SD)	75	(±13)	62	(±6)	69	(±12)
Education, n (%)						
Intermediate vocational education	5	(71)	3	(60)	8	(67)
Higher vocational education	1	(14)	1	(20)	2	(17)
University	1	(14)	1	(20)	2	(17)
Spouse present at interview, yes, n (%)	3	(43)	0	(0)	3	(25)
Living situation, n (%)						
Nursing home	1	(14)	4*	(80)	5	(42)
Independently, with others	5	(71)	1*	(20)	6	(50)
Independently, alone	1	(14)	0*	(0)	1	(8)
Primary diagnosis, n (%)						
Cancer	6	(86)	1*	(20)	7	(58)
Cerebral infarction	0	(0)	2*	(40)	2	(17)
ALS	1	(14)	0*	(0)	1	(8)
Parkinson	0	(0)	1*	(20)	1	(8)
Heart failure	0	(0)	1*	(20)	1	(8)
Year of diagnosis, n (%)						
1992	1	(14)	1*	(20)	2	(17)
1998	0	(0)	1*	(20)	1	(8)
2006	0	(0)	1*	(20)	1	(8)
2016	1	(14)	1*	(20)	2	(17)
2018	4	(57)	0*	(0)	4	(33)
2019	1	(14)	1*	(20)	2	(17)
Deceased, yes, n (%)	NA	NA	3*	(60)	NA	NA
Interview duration, minutes, mean (range)	80	(43-104)	96	(68-125)	87	(43-125)

ALS: Amyotrophic Lateral Sclerosis; NA: Not Applicable; SD: Standard Deviation.

*Information concerning the patient receiving palliative care.

### Essential Elements of Palliative Care

Initially, all participants stated to be satisfied with the care provided and were hesitant in sharing of and reflecting on undesired experiences. Undesired situations often stemmed from inappropriate interactions with specific HCP. Participants linked these experiences primarily to inadequate education/training, workload, staff and financial shortage, and bureaucracy.

Three essential elements and contributing factors of palliative care emerged from the data: **(1) be seen**, **(2) information needs**, and **(3) ensuring continuity**. Illustrative quotes are depicted in Table 2. For all three essential elements tension was identified: a feeling of control/safety vs the feeling of no control/safety, which manifests either in the presence or absence of an essential element.

**Table 2.** Quotes per Identified Theme.

Theme	Respondent	Quote
Be Seen		
Personal attention	#2, man, primary care	‘… I’ll explain it differently…I’ll just take you for example. Suppose you came here and you had to help someone to bed, and you were working the entire evening shift, and you had phones, buttons, or calls in your ear. I don’t need to say more about it…Yeah, how should I say it, I prefer not to see her.
	#11, women, primary care	‘Someone who just comes and sits with you, and I’m very difficult to draw blood from. And yet, they say, “I’ll warm your arm a bit because maybe it’ll work this time.“ you know. And those are just two sentences, but because of that, you do become relaxed… Oh, I think that’s the thing, those small things that are actually quite significant.’
	#5, daughter, nursing home	‘I think the physical aspect is, of course, important, but I find the well-being the most important. In health care. The well-being of the person. There’s a person behind it. It’s not just a thing that you wash and dress quickly, and you’re done. And you know, it’s really a person, and I find that very important, the person behind the resident or the patient. Because then the well-being of the people is also at risk because there is no time left for a moment to sit, have a chat, which is very important, of course. Because the dignity diminishes a bit…’
Alignment of who the patient is as an individual	#11, women, primary care	‘But all of that, I think, is very individual; it’s crucial to consider who you have in front of you.’
	#6, daughter, nursing home	‘I also mentioned that she likes to wear a skirt on Sundays, that she wants to go to church, and that she eats a bit of everything...Well, okay, it’s in the report, I mentioned it during the last multidisciplinary meeting. It looks fine on paper, but the execution, I sometimes find it lacking.’
Feeling connected with HCP	#1, women, primary care	‘He has been the GP here for a very long time, also for my husband. He also accompanied the death of his wife. So, he knows the family situation.’
	#5, daughter, nursing home	‘And the good thing is, those girls know my mother too. Yes, it’s very easy to discuss things with them, and they brainstorm with you.’
Information needs		
Illness trajectory	#3, men, primary care	‘And at the beginning, I didn’t have any issues with her [nurse specialized in palliative care], but at some point, she started talking about, well, the end of life or something like that. And I wasn’t ready for that at all, because I didn’t have any end-of-life concerns, and uh…I said, “Yes, maybe I acted a bit strangely towards you, but it felt a bit odd to me.” at that moment, I didn’t know what the purpose of that was…if something like that comes into view, then I would bring it up. And I know that I can bring it up, and she is there for it.’
	#9, daughter, nursing home	‘It is not always preventable, in any case, but with clear information beforehand. And involve the caregiver [relatives] as much as possible
Multidimensional symptoms And concerns	#1, women, primary care	‘[name nurse specialist in palliative care], she really asks probing questions, and she makes you think. Sometimes, I have things that, well, I’ve never thought about, but she kind of forces you to think about them and form an opinion. I Appreciate that about her. What are your future expectations? What do you expect from that? How are your feelings?” Yeah, those kinds of questions.’
	#12, daughter, nursing home	‘They actually look way too little. Because when we finally got [name nurse specialist in palliative care] from the hospice, the palliative nurse. Then it just went bam bam bam very quickly, and yes. And that was also just great for the caregivers. Once she was in the picture. And when [name nurse specialist in palliative care] came, then you could discuss that, and she could explain it much better. That was a relief for us. Also, if there was something that made you think, “this just can’t be,” you only had to consult, and then she would handle it.’
Ensuring continuity		
A point of contact	#1, women, primary care	‘Yes, I keep returning to her, which is truly a kind of anchor, you might say. And the general practitioner is also a trusted person, but you only engage with them when it’s absolutely necessary, not for casual conversation or such. Of course, I can discuss anything that bothers me with [name specialized nurse], and I can call her if there’s something on my mind. And should there be anything that needs to be done or arranged, she takes care of it.’
	#6, daughter, nursing home	‘Yes, that might sound rather blunt, but that’s how it is. One day it’s (name nurse assistants), and the next day it’s (name nurse assistants), and that’s it. So, I know, by now, I’m familiar with all of them. Thus, I know where to go.’
Availability of HCP	#2, man, primary care	‘No, not at the moment. I Feel very well, and everything is well-organized. I Know that if there’s something, all I have to do is make a call, and they’ll come right away, so yes.’
	#12, daughter, nursing home	‘You know, it’s all very cumbersome. It’s like this continuous process. And then, even after that, they had to increase it again, and the caregivers had to consult with the general practitioner. And if the general practitioner wasn’t available, especially in the evenings, it would be a replacement. So, you see, that’s where we encountered quite a challenge.’
	#9, daughter, nursing home	And isn’t it simply the most important thing that there are short lines of communication?
Coordination of care	#3, men, primary care	‘And then one of the ladies said, “I’ll take care of it, I’ll discuss it with the GP when she’s at the GP on that Wed.” but it often takes a long time. After two months, I haven’t heard anything, and then she was here again. She says, “how is it with the medication?” “which one?” I ask. “Haven’t you received it yet?” she says. “Well,” I reply, “that’s absurd. I’ll call right away.” two days later, I’m called by the pharmacy to schedule a meeting to discuss how I want it and such and so forth.’
	#9, daughter, nursing home	‘My mother had pressure sores, and she also had a special mattress, etc. She had been to the hospital for an examination, and they recommended a particular plaster. However, here, they said, ‘we don’t use that; we always use this instead.' so, there were occasional points of disagreement, such as a certain ointment for her feet that had to be used, and they said, ‘well, we’re not familiar with that; we have this, and it’s just as good.' Then, I would consult with the general practitioner, who would then get in touch with the specialist. So, it didn’t always go smoothly.’
	#6, daughter, nursing home	‘Yes, and good guidance for those individuals, meaning they may have completed an education or training, but they need proper guidance. That way, you have competent caregivers in health care, which is truly essential. It’s also about improving coordination among them… Sometimes I say something to one person, and then I say it to another, and the other person is unaware of it,’

#### Essential Element 1: Be Seen

The most outstanding essential is ‘be seen’, which refers to the feeling of being seen as a person rather than the illness through personal attention, alignment to who the patient is as a person and feeling connected.

#### Personal Attention

All participants expressed a need for empathy. The HCPs’ attitude and communication competencies are vital. Further, this need manifests in HCPs being present in the moment, listening attentively, and conveying that they are not in a hurry. This is crucial in routine care but extends to engaging in conversations, sitting with the patient, stopping by, and remembering details previously discussed. Participants value HCPs who exhibit warmth, empathy, understanding, and genuine interest, and make patients feel that their concerns matter, taking them seriously and treating them as equals in the conversation.

#### Alignment to Who the Patient is as an Individual

It is crucial for participants that care is tailored to the individual and aligns with the patient’s identity. To achieve this, HCPs must delve into the person behind the patient. This involves knowing the patient’s preferences, such as their food and music choices, their interests, and their personality. When this personalized approach is not integrated into care and systematically incorporated, participants feel that their autonomy and voice are not respected.

#### Feeling Connected with HCPs

Participants emphasized on the importance to feel connected with the HCPs by developing a relationship that goes beyond a purely professional connection and involves a genuine bond where they know each other well. This connection impacts the participants' emotional state, trust, and the quality of interactions. A consistent team of HCPs contributes to fostering these personal connections.

#### Essential Element 2: Information Needs

Information needs refers to the need for a proactive approach with means of information on illness trajectory and multidimensional symptoms and concerns.

#### Illness Trajectory

Participants require comprehensive information about their illness trajectory, by means of professional expertise, clear expectations for different scenarios, explanations for the care process, and guidance on what questions and wishes they can express. It is essential for this guidance to commence from the outset of the trajectory and to adapt continuously to the needs and preferences of the patient or relative. Conversations about illness, dying, and death often do not occur until the patient’s condition deteriorates or the terminal phase is identified and is influenced by the patient’s or relative’s ability to accept the illness. Relatives require additional guidance from HCPs on how to support their loved ones through the illness trajectory. This includes information about what is considered normal, how the illness presents itself, and what the dying process entails.

#### Multidimensional Symptoms and Concerns

Participants, especially relatives, require HCPs who can anticipate and outline scenarios for potential future care to prevent symptoms and undesirable situations. Moreover, participants highly value clarity regarding symptoms and interventions, leveraging the expertise of HCPs. This need intensifies as the patient’s condition deteriorates. In most cases, participants experienced a reactive HCP approach, with some being proactive by asking questions. Most HCPs have conversations with participants about advance directives but have less attention to additional proactive care agreements.

Furthermore, the emphasis of care is primarily placed on physical symptoms, aligning with participants' expectations. Support for psychological symptoms, and social and spiritual concerns is often sought within the family. One patient mentioned an HCP who monitored symptoms over time using a symptom diary, which was highly appreciated by the patient.

#### Essential Element 3: Ensuring Continuity

The last essential element for patients and their relatives is ‘ensuring continuity’ which refers to organizational aspects of care: single point of contact, availability of HCP, and coordination of care.

#### Single Point of Contact

All participants value a dedicated contact person to guide them through the course of illness. This professional, whom they trust and can confide in regarding their symptoms, concerns, and fears, should have palliative care expertise and be available to answer questions, provide support at every stage of the illness, and aid in preparing for future care. Most participants could not identify a contact person.

#### Availability of HCPs

The availability of HCPs means that participants can always reach out to an HCP, whether by phone, email or in person, for questions or consultations. Most participants appreciate regular consultations, even when the patient’s condition is stable. In addition, undesired situations are experienced with fluctuating personnel. Most participants expressed the need for more frequent contact with physicians or nurses who can coordinate care and treatment. Furthermore, the presence HCPs who are qualified to make informed decisions becomes particularly important as the illness progresses and becomes unstable.

#### Coordination of Care

Participants value HCPs support for practical aspects and stress the need for improved collaboration and communication across health care settings. They experienced issues in hospital-district teams and or GP and hospital-nursing home communication, mainly due to information loss and care plan disagreements. Internal collaboration challenges included inconsistent care plan communication, insufficient information during patient transfers, and unclear roles and responsibilities.

## Discussion

This study explored the palliative care experiences of patients with a life-limiting illness at home and in a nursing home and their relatives. Three essential elements of palliative care emerged: **(1) be seen**, **(2) information needs**, and **(3) ensuring continuity**. In each essential element, a tension was identified that manifests in patients and relatives: feeling control/safety vs feeling a loss of control/safety. Loss of control and safety emerged if these essentials were not met and depended largely on the individual HCP.

Patients and relatives in both settings perceived the same essential elements of palliative care. In a study on barriers to providing palliative care for patients at home or in nursing homes with HCPs we found that barriers differed within the settings; in primary care, mainly organizational barriers were identified whereas, in nursing homes, most barriers were related to care content.^37^ ‘Be seen’, ‘information needs’, and ‘ensuring continuity’ seem to be preconditions for patients to live their lives, regardless of the environment, given the conditions of the palliative phase.

Our study highlighted the importance of how HCPs provide care. Undesired experiences were largely influenced by a specific HCP. As a result, patients depend on luck when it comes to the HCP attending to them at their bedside.^38^ HCPs effectiveness can be shaped by care organization constraints like workload and staff shortages. However, it goes beyond time; patients and relatives acknowledge these challenges. The key factors lie in the attitudes and competencies of HCPs. To see a patient as a unique person stood out the most. Several other studies confirmed this importance. Studies in home care showed that palliative care patients preferred care that supported to ‘be seen as the person they had always been’,^3^ and focus on the person was an element that patients and relatives considered essential.^21^ Another study under palliative care patients in home, nursing homes and hospitals and their relatives showed that a ‘death in which one could maintain a connection with one’s previous identity’ was described a good death.^39^ A conceptual framework to understand the patient experience, sets the person across the whole continuum, indicating that the patient and user of health care services is the same unique person they have been.^40^ Other studies with patients in the palliative phase in various settings support our results as they identified the importance to feel safe in the place of care,^41^ that palliative care focused on living a meaningful life,^13^ and that palliative care included respect and empathy.^42^ Furthermore, showing empathy, which involves perceiving a patient as a unique individual, and providing open and honest information is essential for effective communication,^43^ which results in turn in favorable outcomes. A study by Westendorp et al showed that information that an HCP conveys with empathy is remembered better than information shared without empathy^44^; which also enhances shared decision-making. Other studies revealed that empathic communication and providing specific prognostic information enables symptom control, guides decision-making and has an immediate effect on how the patient feels, on the satisfaction of patients, feelings of insecurity and fear, on the trust they have in HCPs, and on the future.^43,45-47^ However, patients often perceive communication with HCPs as ineffective.^48-50^ In our study on barriers in primary care and nursing homes, HCPs did not perceive barriers in communication with patients and relatives, which exposes the necessity of communication.^37^ In addition, all three essential elements help HCPs to shape proactive palliative care. Patients and relatives who feel seen as a person, receive clear and honest information, and who have a single point of contact and HCPs that are available, may as well be more open about their symptoms and concerns, may be more willing to engage in conversation about (future)care needs, death and dying, ^51^ know what to expect which helps them to make well-considered decisions and positively influences symptom management.

### Strengths and Limitations

A strength of this study is the inclusion of participants from both primary care and nursing home settings, providing a comprehensive understanding of essential palliative care elements among patients and relatives. Participants varied in gender, education, patients’ place of residence and patients’ diagnosis, providing a presentation of various experiences among a diverse group. The diversity in age was limited; younger patients might have other essential elements that are important to them due to an active family life and younger children. Mainly primary care patients and nursing home relatives were interviewed due to contextual constraints. Participants tended to express satisfaction with care rather than undesired experiences. Efforts to explore these experiences were challenging, possibly due to care dependency. A common strategy involved presenting a hypothetical undesired situation, prompting participants to share their own experiences. This strategy resulted in additional information on undesired situations, which was not spontaneous.

### Implications

This study highlighted that the essence of palliative care is not always optimal. HCPs must be aware of their impact on patients' and relatives’ well-being simply on how they engage and communicate with them. Experiences of patients and relatives are highly influenced by their interactions with HCPs. An empathic attitude and competencies, especially on communication, are important in providing palliative care. To support HCPs in daily practice, education and training should focus on communication skills, with a special endorsement for nursing staff given their extensive interaction with patients and relatives. Further, incorporating an increased use of PROMS, such as the Utrecht Symptom Diary four dimensional (USD-4D),^52,53^ could help HCPs in their communication with patients and relatives and to really put the values, wishes and needs first.

## Conclusion

Patients living at home and in nursing homes, along with their relatives, expressed the same essential elements of palliative care that matter to them. They emphasized the importance of being recognized as a unique person beyond their patient status, receiving honest and clear information aligned with their preferences, and having care organized to ensure continuity. HCPs should be aware of these essential elements and should use these insights as guidance for daily practice. Adequate competence and skills are needed, together with a care organization that enables continuity in order to provide safe and person-centered care.

## Supplemental Material

Supplemental Material - Being Seen as a Unique Person is Essential in Palliative Care at Home and Nursing Homes: A Qualitative Study With Patients and Relatives Supplemental Material for Being Seen as a Unique Person is Essential in Palliative Care at Home and Nursing Homes: A Qualitative Study With Patients and Relatives by Katrin Kochems, Everlien de Graaf, Ginette M. Hesselmann, and Saskia C. C. M. Teunissen in American Journal of Hospice and Palliative Medicine®
