# Supplementary material for: Being Seen as a Unique Person is Essential in Palliative Care at Home and Nursing Homes: A Qualitative Study With Patients and Relatives
Source: Am J Hosp Palliat Care. 2024 Apr 6;42(2):207–16. doi: 10.1177/10499091241242810 (PMC11636018; doi:10.1177/10499091241242810)
Supplement: Supplemental Material - Being Seen as a Unique Person is Essential in Palliative Care at Home and Nursing Homes: A Qualitative Study With Patients and Relatives [file sj-pdf-1-ajh-10.1177_10499091241242810.pdf]

Supplementary material 1. Topic list

Topic	Open question	Sub-question
**Experienced care**	Looking back at your last week:<br>What was your last week like in terms of the care you received?	Do you feel you need little or a lot of care?
	Does the care you need match your needs/wishes and symptoms/concerns?	What were the moments when you were very satisfied with the care provided? (what difference did this satisfaction make?)
	What does care mean to you?	What went well in recent weeks and why?
		What didn't go well in recent weeks and why?
**Identification of multidimensional symptoms and concerns**	If you look at your wishes/priorities:<br>What are you busy at the moment?	Is it clear who the point of contact is for care and other questions?
	Is attention paid to your wishes, needs, symptoms/concerns?	Do you also discuss psychological, social or existential issues with your HCPs? (examples)<br>- If yes, with whom?<br>- If no. do you discuss this with other people?
		Does the HCP speak to you about changing situations?
		Do the HCPs recognize what is important for you?
		Do HCPs ask about your wishes and needs? Is it being explored what this means for you?
		Do you feel seen and known by HCPs? Do you feel safe reporting needs, symptoms, and concerns?
		What were moments when recognizing your wishes and needs went well? What does that mean?
**Decision-making**	Do you feel you have enough say in decisions about your care/treatments?	Do you want to make your own decisions regarding your own care?<br>- Do you think that is important?<br>- What do you mean by making your own decisions?<br>- Can you discuss with healthcare providers whether and how you want to make a decision?<br>- Is there attention to make you more assertive?
	Do you still have your own control, and what does that look like in your eyes?	Do healthcare providers explain all care and treatment decisions made to you?<br>- Are decisions clear?<br>- Are you satisfied with the decision-making about your treatment and care?
		- Are your wishes and priorities included in the care plan?
		Do you feel understood/respected?<br>Do you have a good relationship with HCPs? (if no, why)
		What goes well in decision-making regarding your treatment and care, and why?<br>What is not going well, and why?
**Communication with HCPs**	How do you experience communication with HCPs?	Was there communication?<br>- What does your ideal communication with healthcare providers look like?<br>- Do you feel heard?<br>- Do your loved ones feel heard?

		- What went well in communication with healthcare providers in recent weeks and why?<br>- What didn't go well, why?
	Is everything covered that you want to talk about? (ups/downs)	Home care: Do the moments of care come at the right time for you, appropriate to the care needed and your daily life?
	Multiprofessional consultations:<br>Will you be told when a nurse coordinates with the doctor, for example?	Are you present at meetings?<br>- Are you asked what is important to you?<br>- Are there discussions before and after?<br>- Who communicates this with you?<br>- Are results always clear to you?<br>- What do you want recorded of your information?<br>- Are you satisfied with the recording of information?<br>- Do you keep a diary yourself?
**Continuity of care**	Is there communication between different HCPs?	Do you have insight into communication?<br>- Do you think that is important?<br>- Do you feel that information about your care is properly exchanged?<br>- What are coordination’s like?
	How do you experience communication between HCPs, for example between the hospital and your GP?	Is there a lot of change in care providers or do you have permanent care providers?
	Confidence in the care you experience?	What's going well? What not? Why?
**The burden on relatives**	What contributes to your ability to handle care?	Do you feel you receive enough support from HCPs?
	Tell me about your most beautiful and most difficult moments.	What is your role in the care of your loved one? Are you satisfied?
		What is going well, and why?<br>What is not going well, and why?
**Care recommendations**	What would the ideal situation look like for you?	What do you need for optimal care?
